# Quantized Analog Inference Hardware Enabled by MoS_2–_Hf_1‐x_Zr_x_O_2_ FeFET Synapses

**DOI:** 10.1002/advs.78366

**Published:** 2026-10-22

**Authors:** Jeongmin Park, Moonyoung Jung, Hyo‐Bae Kim, Chanhyuk Park, Seohyun Lee, Ki Kang Kim, Hongkee Yoon, Ji‐Hoon Ahn, Dongseok Suh

**Affiliations:** ^1^ Department of Physics Ewha Womans University Seoul Republic of Korea; ^2^ Department of Energy Science Sungkyunkwan University Suwon Republic of Korea; ^3^ Department of Materials Science and Chemical Engineering Hanyang University Ansan Republic of Korea; ^4^ Department of Semiconductor Physics Kangwon National University Chuncheon Republic of Korea

**Keywords:** ferroelectric field‐effect transistors, hafnium zirconium oxide, monolayer MoS_2_, neuromorphic computing, quantized neural networks

## Abstract

Analog in‐memory computing can accelerate edge‐AI inference by executing vector–matrix multiplication directly in memory crossbars, but practical analog deployment is constrained by finite weight resolution and stochastic programming. Here we demonstrate program‐pulse‐engineered MoS_2–_Hf_1‐x_Zr_x_O_2_ (HZO) ferroelectric field‐effect transistor (FeFET) synapses and a hardware‐consistent quantized machine‐learning workflow for noise‐robust analog inference. The device integrates an atomically thin monolayer MoS_2_ channel, representing ultimate body‐thickness scaling in advanced logic roadmaps, with a CMOS‐compatible HZO ferroelectric gate stack. Operating in a near‐coercive regime, trains of identical potentiation/depression pulses yield gradual and reversible multi‐level conductance updates with ∼100 states, enabling array‐realistic programming without per‐device program‐and‐verify. Systematic pulse engineering provides knobs to tune update granularity and dynamic range, and enables offset control of the usable conductance window via asymmetric depression conditions. Crucially, we quantify cycle‐to‐cycle write variability using state‐dependent Δ*G* statistics and incorporate the measured quantization levels and noise directly into training and evaluation. Using MNIST as a benchmark, noise‐aware quantization‐aware training outperforms post‐training quantization across quantization levels and noise scaling, sustaining up to ∼97% test accuracy under low precision and elevated noise. These results connect measured programming physics to robust quantized analog inference and establish practical device–algorithm co‐design principles for scalable FeFET‐based synaptic hardware.

## Graphical abstract

A monolayer MoS_2_–HZO FeFET is presented as a pulse‐engineered neuromorphic device suitable for crossbar‐based analog computing. Programming‐pulse design enables tunable conductance trajectories for synaptic weight updates, while quantized machine‐learning approaches account for device variation, nonlinearity, and write noise, translating analog hardware constraints into robust and energy‐efficient inference for edge AI.

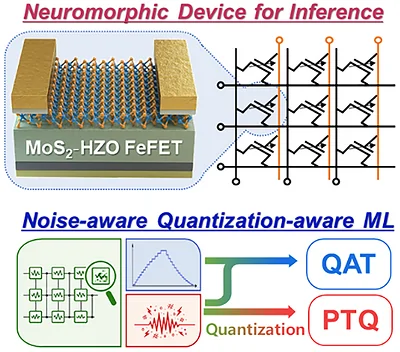

## 1 Introduction

Artificial neural networks rely on large numbers of tunable weights, and mapping these weights directly onto memory devices can accelerate the vector–matrix multiplications (VMMs) that dominate inference [1, 2, 3]. Analog in‐memory computing (IMC) implements VMM in a single physical step by applying an input‐voltage vector to crossbar word lines and summing the resulting currents on bit lines [4, 5]. However, deployable analog IMC must confront two inseparable hardware realities—finite weight resolution and stochastic weight updates—because conductance states are discrete and programming is noisy [6]. Bridging device‐level behavior to system‐level accuracy therefore requires not only a programmable synaptic device, but also a modeling and training strategy that remains faithful to how arrays are actually written and read.

Ferroelectric field‐effect transistors (FeFETs) are attractive synaptic devices because they offer voltage‐controlled, non‐volatile conductance modulation in a three‐terminal geometry that separates read and write [7, 8, 9]. CMOS‐compatible HfO_2_‐based ferroelectrics such as Hf_1‐x_Zr_x_O_2_ (HZO) are especially compelling: partial polarization switching can generate intermediate states when pulse amplitude and duration are engineered [10, 11, 12, 13, 14]. Yet the key scalability question is not simply whether intermediate states exist, but whether they can be reached predictably with identical pulses across many synapses. Adaptive or incremental‐pulse schemes can improve linearity on a single device, but they typically require program‐and‐verify feedback and per‐synapse state tracking, which becomes costly and slow at crossbar scale [15].

Write noise further complicates array‐level deployment [16]. Even when identical pulses are used, the conductance change per pulse can vary cycle‐to‐cycle, creating weight uncertainty that can degrade inference and silently invalidate performance projections if noise is averaged out. From a machine‐learning viewpoint, this is tightly linked to quantization: a finite set of conductance states maps naturally onto a quantized neural network. Quantized machine learning (QML) therefore provides a principled language for analog synapses, but only if the quantization levels and noise statistics are derived from measured device behavior. In this context, post‐training quantization (PTQ) is a convenient baseline, whereas quantization‐aware training (QAT) offers a route to robustness by optimizing the model under the same discrete and noisy constraints expected at deployment [17, 18, 19, 20].

Here we present a program‐pulse‐engineered synaptic platform based on a monolayer MoS_2–_HZO FeFET that directly targets identical‐pulse programmability and hardware‐consistent inference [21, 22, 23]. Crucially, monolayer MoS_2_ is not introduced merely as another 2D semiconductor, but as an ultimate‐scaling channel with an ultrathin body that enables the strongest electrostatic gate control in the thickness limit [24, 25]. This “ultrathin‐body” paradigm is a key direction in semiconductor roadmaps for continued scaling, and it provides a natural materials foundation for aggressively scaled transistors and dense synaptic arrays where gate efficiency and variability control are paramount [26, 27]. In our platform, this ultimate‐thin MoS_2_ channel is paired with HZO, whose CMOS compatibility makes it a practical ferroelectric for integration with scaled transistor technologies. Together, the MoS_2_ ultrathin‐body channel and the CMOS‐compatible HZO ferroelectric gate stack form a scaling‐relevant synaptic transistor platform in which polarization–channel coupling can be leveraged for multi‐level, non‐volatile weight storage. Recent studies have demonstrated 200‐mm‐wafer‐scale MoS_2_ transistor integration using industry‐compatible fabrication processes [28] while ALD‐grown HZO ferroelectric capacitors have shown electrical uniformity across 12‐inch Si wafers [29]. These advances support the process‐level feasibility of large‐area MoS_2–_HZO device integration.

We connect device behavior to inference using two complementary, hardware‐realistic evaluation tracks. First, a device‐informed crossbar simulation maps neural‐network weights directly onto measured potentiation/depression (P/D) trajectories while explicitly retaining cycle‐to‐cycle write fluctuations, avoiding over‐optimistic accuracy projections caused by noise averaging. Second, a quantized machine‐learning framework incorporates device‐derived quantization and write‐noise statistics to benchmark PTQ against noise‐aware QAT across precision and noise regimes, identifying the training strategy required for robust deployment on inherently quantized, noisy analog synapses.

In this work, we make the following contributions:

- **Array‐compatible synaptic programming**: We demonstrate an identical‐pulse, near‐coercive programming regime in a monolayer MoS_2–_HZO FeFET that yields gradual, multi‐level potentiation/depression with reproducible trajectories suitable for crossbar‐scale operation.
- **Pulse‐engineering design knobs**: We establish a practical pulse‐engineering methodology that tunes effective weight range and update granularity by adjusting pulse amplitude and width, and further provides an offset control via asymmetric depression conditions—enabling “on‐demand” shaping of the usable conductance window without changing the device structure.
- **Noise as a measurable, modelable constraint**: We quantify cycle‐to‐cycle write variability using state‐dependent *Δ*G statistics (capturing the distribution of conductance updates rather than averaging them out) and incorporate these noise‐inclusive trajectories into device‐informed simulations to avoid idealized performance estimates.
- **Hardware‐consistent quantized inference**: Using device‐derived quantization and write‐noise constraints, we benchmark PTQ against noise‐aware QAT and show that QAT preserves inference accuracy far more effectively in low‐precision and noisy regimes, establishing a practical co‐design rule for deploying quantized, noisy analog synapses.

## 2 Results and Discussion

### 2.1 Analog Crossbar Inference and Quantized Synapses

To connect the MoS_2–_HZO FeFET device to neuromorphic computation, we first outline the conceptual framework summarized in Figure 1a. A fully connected neural network for MNIST digit classification can be mapped onto a FeFET‐based synaptic array (crossbar‐like), where each synaptic weight is represented by the device conductance and vector–matrix multiplication (VMM) is implemented by current summation during inference [30]. In this picture, every conductance state of an individual FeFET directly corresponds to a weight in the neural network.

**FIGURE 1:**
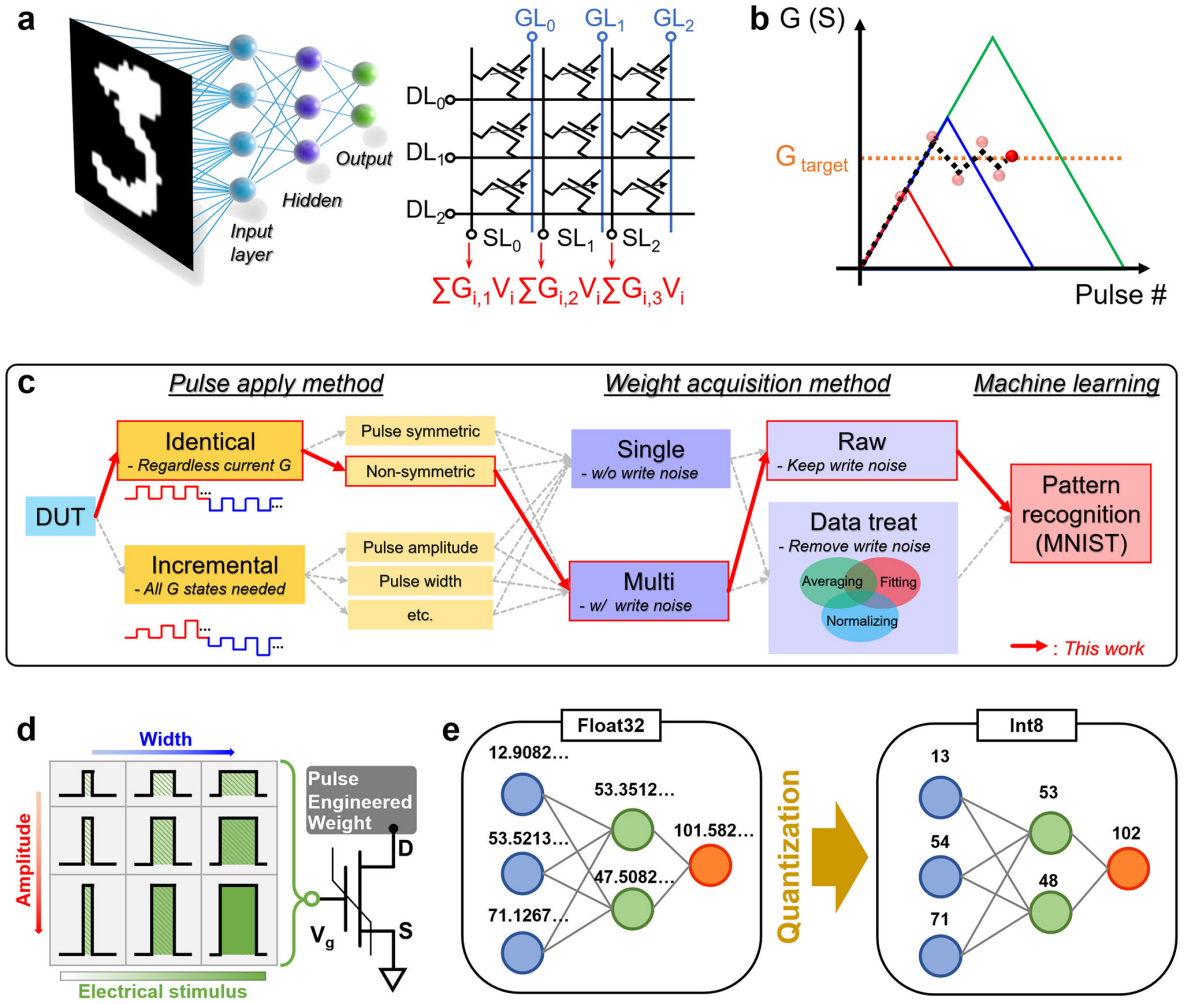
Concept of analog crossbar inference and quantized neural‐network modeling for the MoS_2_–HZO FeFET synapse. (a) Fully connected MNIST network and its realization by an FeFET crossbar, where each device conductance encodes a synaptic weight and vector–matrix multiplication (VMM) is performed in one analog step. (b) Conceptual illustration of iterative potentiation/depression pulses driving the device conductance toward a target value in the presence of write noise. (c) Flow chart comparing pulse‐programming schemes and highlighting the identical‐pulse operation used in this work: extraction of device‐consistent weight trajectories including write noise, and direct use of these weights in MNIST recognition. (d) Pulse‐engineered weight update scheme, where tuning the gate‐pulse amplitude and width controls the electrical stimulus, thereby modulating updates of the synaptic weight represented by the channel conductance and reshaping the resulting P/D trajectories. (e) Schematic of weight quantization from Float32 to a finite set of levels, highlighting the need for QML when mapping neural networks onto analog FeFET hardware.

Programming such an array amounts to steering each device conductance toward a desired target value by applying sequences of potentiation/depression (P/D) pulses. As sketched in Figure 1b, repeated pulses move the conductance up or down in small increments, and the trajectory fluctuates around an ideal path because of cycle‐to‐cycle write variability (and, more generally, array‐level variability). This process is conceptually analogous to iterative weight updates in digital neural‐network training, except that here the “update rule” is implemented physically by gate pulses and partial ferroelectric switching rather than numerically in software [31].

Our synaptic device measurement protocol is summarized in Figure 1c, while Figure 1d illustrates the pulse‐engineering concept: tuning the gate‐pulse amplitude and width controls the effective electrical stimulus, thereby modulating synaptic weight updates and reshaping the resulting P/D trajectories. In all synaptic experiments, once a programming condition was selected, we operated the device with trains of identical programming pulses applied to the gate, so that each update step corresponded to the same nominal electrical stimulus; this operating mode matches practical array operation, where many synapses must be addressed in parallel and it is not feasible to tailor pulse conditions for each device and its history. An alternative incremental‐pulse scheme can in principle improve linearity for a single device but typically requires per‐synapse state tracking and feedback (program‐and‐verify), which becomes costly at array scale [32]. We therefore focused on identical‐pulse operation and extracted synaptic weights by repeatedly cycling the device through P/D while explicitly retaining the cycle‐to‐cycle conductance fluctuations (write noise) rather than averaging them out. These noise‐inclusive conductance trajectories were then used directly in our neural‐network simulations, providing a device‐informed model that more closely captures how an analog FeFET synaptic array would behave in practical neuromorphic hardware.

From the viewpoint of machine learning, the hardware effectively implements quantized weights rather than ideal 32‐bit floating‐point values. This motivates a quantized machine‐learning treatment of the FeFET synapse: the device's finite conductance levels are treated as the allowed weight states, and training procedures such as post‐training quantization (PTQ) and quantization‐aware training (QAT) are used to obtain models that match the hardware constraints (Figure 1e). In the remainder of this section, we move from the device structure and basic ferroelectric properties (Figure 2), through synaptic operation and pulse engineering (Figure 3 and Figure 4), to system‐level quantized neural‐network behavior (Figure 5).

**FIGURE 2:**
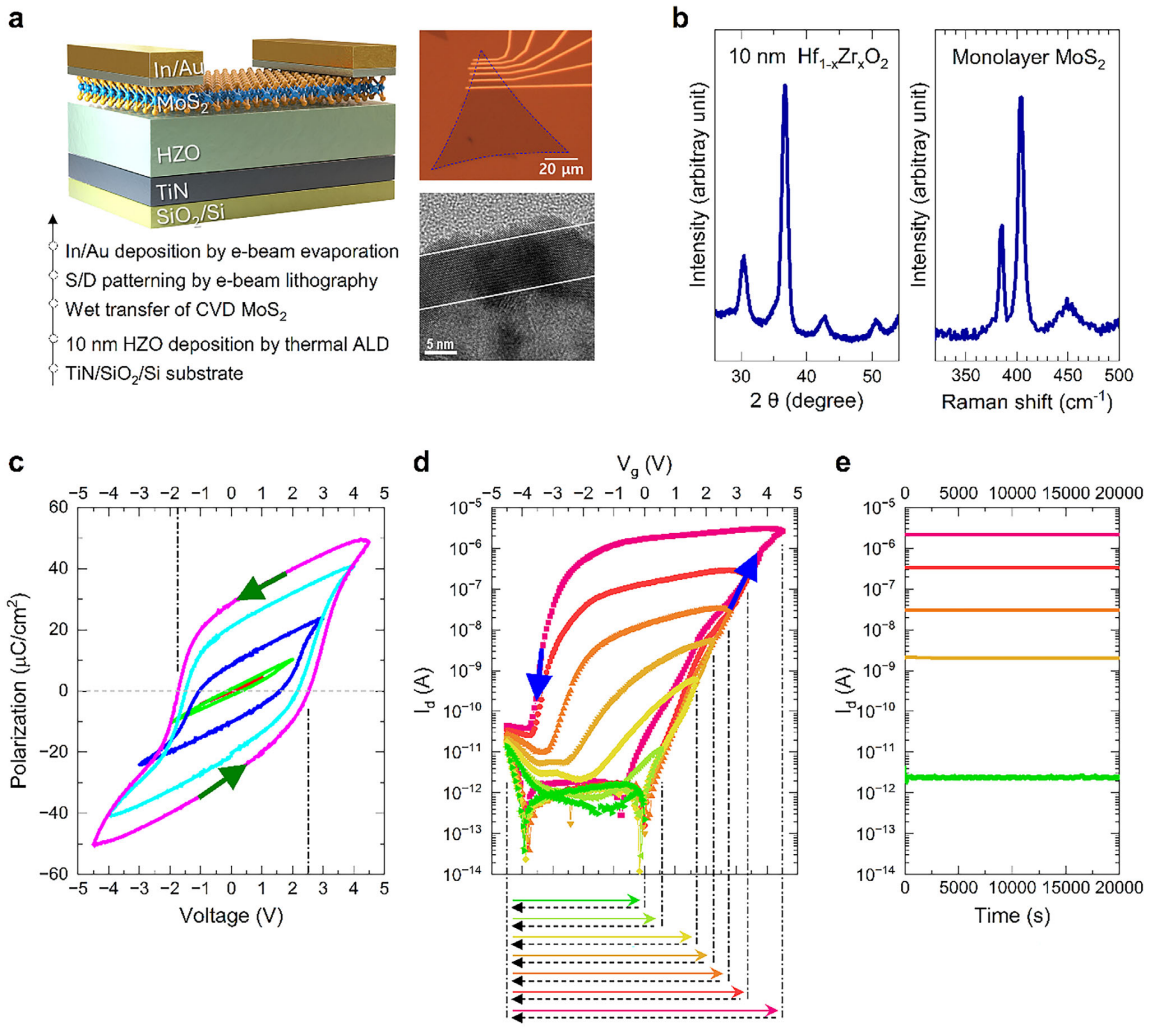
Device structure and basic ferroelectric memory characteristics. (a) Device stack and fabrication sequence of the bottom‐gated monolayer MoS_2–_HZO FeFET. (b) XRD of the 10 nm HZO film confirming the orthorhombic ferroelectric phase, and Raman spectrum of CVD MoS_2_ confirming a monolayer channel. (c) P–V loops of an Au/In/HZO/TiN capacitor showing clear ferroelectric hysteresis with coercive voltages around +2.5 V and −1.7 V, slightly shifted to positive bias. (d) Asymmetric *I*_d–_ *V*_g_ sweeps of the FeFET exhibiting counter‐clockwise ferroelectric hysteresis; the onset of the memory window matches the capacitor coercive voltages and yields an ON/OFF ratio of ∼10^5^–10^6^. The measured device has a channel width 6.5 µm and length 1 µm. (e) Retention of multiple programmed conductance states for at least 2 × 10^4^ s at V_g_ = 0, demonstrating robust non‐volatile behavior suitable for inference‐oriented analog AI hardware. The device was initialized with a −4.0 V gate pulse, and distinct conductance states were programmed using +2.0, +2.5, +3.0, and +3.5 V gate pulses with a pulse width of 200 ms.

**FIGURE 3:**
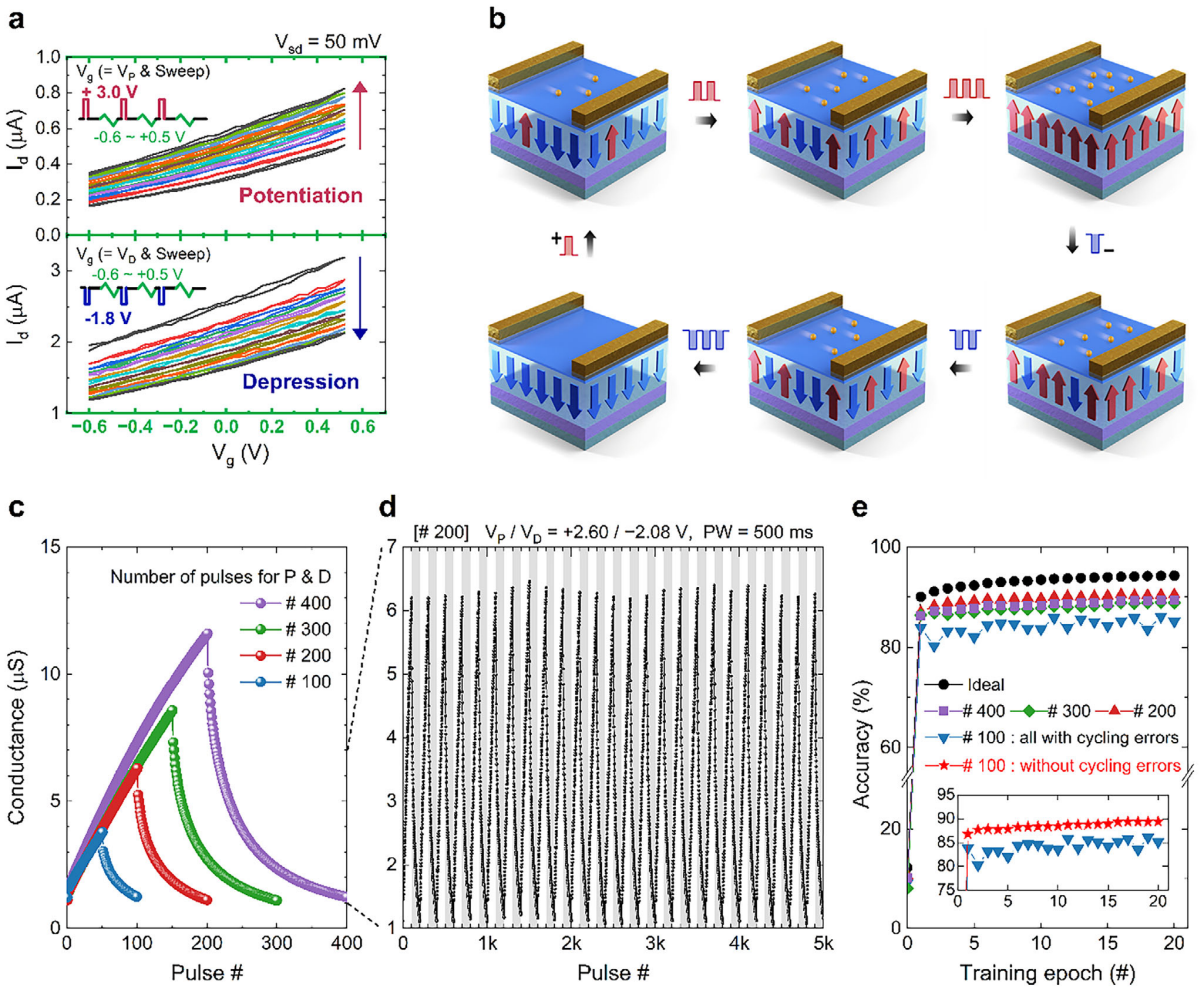
Synaptic P/D characteristics and device‐informed crossbar simulation. (a) Near‐coercive *I*_d–_ *V*_g_ measurements after repeated +3.0 V (potentiation) and −1.8 V (depression) pulses with a pulse width of 200 ms, showing gradual conductance changes while the transfer characteristics were measured within a narrow *V*_g_ window to avoid additional ferroelectric switching during readout. (b) Schematic of partial HZO polarization switching that produces reversible potentiation and depression of the MoS_2_ channel conductance. (c) P/D curves for different pulse numbers (#100–400) under fixed P/D pulse conditions (identical pulses within each polarity), showing self‐similar scaling of loop shape and an increasing number of accessible levels. (d) Cycling endurance for the #200 condition over 5000 pulses, confirming stable analog programmability with minimal drift. (e) Crossbar‐level MNIST simulations using measured P/D characteristics, where the #200 case yields the best balance between dynamic range and accuracy, closely approaching the ideal digital baseline. The inset compares simulations with and without cycling‐induced write noise for the #100 case, revealing that neglecting write noise leads to overly optimistic accuracy estimates.

**FIGURE 4:**
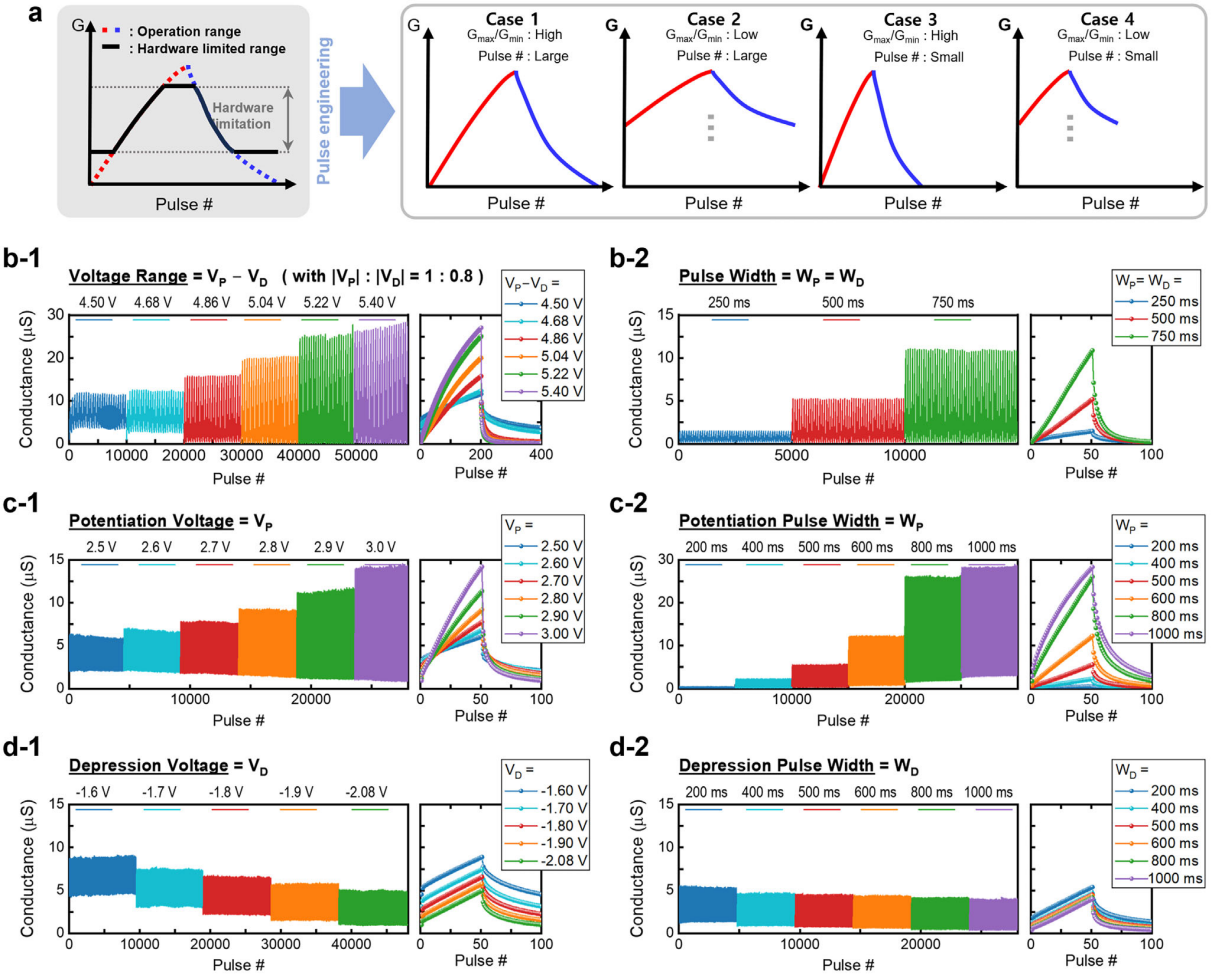
Pulse‐engineered control of P/D curves and effective weight resolution. (a) Conceptual design map linking dynamic range (G_max_/G_min_) and number of levels (Pulse #) to hardware limits, and four representative operating regimes that can be selected by tuning programming conditions. (b‐1, b‐2) Simultaneous modulation of potentiation and depression strength: increasing pulse amplitude (b‐1) or width (b‐2) enlarges the dynamic range but makes the conductance‐level granularity coarser for a fixed pulse count. (c‐1, c‐2) Potentiation‐only tuning: varying the potentiation amplitude (c‐1) or width (c‐2) strongly reshapes the overall P/D loop, indicating that potentiation strength primarily sets the dynamic range and level density. (d‐1, d‐2) Depression‐only tuning: changing depression amplitude (d‐1) or width (d‐2) mainly shifts the P/D loop vertically, adjusting the conductance offset while keeping the dynamic range nearly constant. Raw P/D cycling traces under each condition are shown alongside the corresponding P/D curves in b–d. Detailed programming conditions are provided in Figure S6.

**FIGURE 5:**
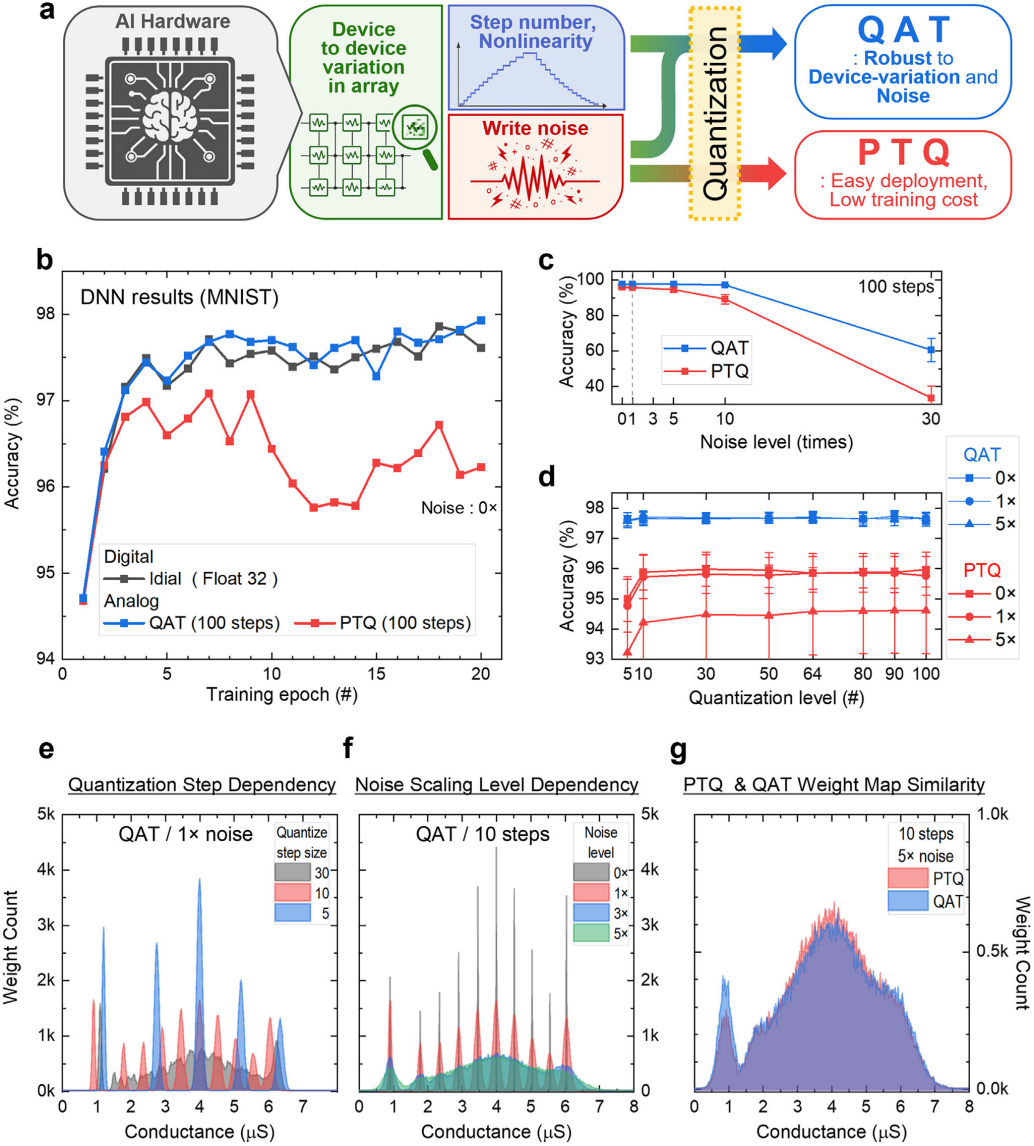
Quantization‐aware modeling of FeFET synapses: QAT vs. PTQ and write‐noise tolerance. (a) Conceptual overview of device non‐idealities in analog AI hardware—device‐to‐device variation, nonlinearity and write noise—and how quantized ML approaches (PTQ and QAT) translate these constraints into robust inference models. (b) MNIST recognition accuracy vs. epoch for an ideal Float32 network and FeFET‐based analog models with 100 quantization levels (100 steps): QAT closely follows the ideal accuracy, whereas PTQ shows lower and more fluctuating performance even in the 0 × write‐noise setting. (c) Accuracy vs. write‐noise scaling (0 ×–30 ×) for 100‐level QAT and PTQ models; QAT remains robust up to ∼10 × noise, while PTQ accuracy degrades rapidly with increasing noise. (d) Accuracy vs. quantization level (5–100 levels) under different noise factors (0 ×, 1 ×, 5 ×); QAT maintains high accuracy across all conditions, whereas PTQ is more sensitive to both coarse quantization and noise. In c,d, data points show the mean accuracy over 35 runs with distinct random seeds, and error bars represent ±1 standard deviation. (e–g) Weight histograms (qualitative comparison). (e) QAT weight distributions at 1× noise for different quantization levels. (f) QAT weight distributions at fixed 10 levels under different noise scaling. (g) PTQ vs QAT weight distributions at 10 levels and 5 × noise, highlighting that similar conductance distributions can still yield different accuracies due to noise‐aware training.

### 2.2 Device Structure and Ferroelectric Memory Properties

The MoS_2–_HZO FeFET is realized on a HZO/TiN/SiO_2_/Si stack, as described in the device schematic and process flow (Figure 2a). A TiN bottom electrode and a 10 nm HZO layer form the ferroelectric gate stack, on top of which a CVD‐grown monolayer MoS_2_ channel is transferred and contacted by In/Au source and drain electrodes. Structural and spectroscopic characterizations are consistent with the formation of a ferroelectric HZO phase and a high‐quality monolayer channel: the grazing‐incidence x‐ray diffraction (GIXRD) pattern of the HZO film exhibits diffraction peaks associated with orthorhombic‐related polar phases of HfO_2_, while the Raman spectrum of the MoS_2_ channel shows the expected separation between the E^1^_2g_ and A_1g_ modes for a monolayer (Figure 2b). Together, these data verify the intended stack of a crystalline ferroelectric HZO layer and an atomically thin semiconductor channel.

Ferroelectric switching in HZO is directly confirmed by polarization–voltage (P–V) measurements on a separate Au/In/HZO/TiN capacitor with the same 10 nm HZO layer (Figure 2c). Clear P–V hysteresis loops are observed as the voltage amplitude is increased, with remanent polarization on the order of several tens of µC/cm^2^. The coercive voltages are approximately +2.5 V and −1.7 V and exhibit a slight shift toward positive bias, indicative of built‐in asymmetry in the ferroelectric stack. Importantly, the voltage range over which polarization switching occurs in this test capacitor closely matches the gate‐voltage range where hysteresis appears in the FeFET transfer characteristics, linking the capacitor behavior to the transistor operation.

Transfer measurements of the MoS_2–_HZO FeFET under asymmetric gate‐voltage sweeps show the expected ferroelectric‐gated transistor behavior (Figure 2d). After presetting the ferroelectric polarization with a negative gate pulse (−4.5 V), the bottom‐gate voltage (applied to the TiN gate) is swept toward positive bias with progressively larger maximum amplitudes. The *I*_d–_ *V*_g_ curves exhibit counterclockwise hysteresis whose width increases with the sweep range, consistent with polarization‐induced threshold shifts in HZO (Figures S3 and S4). A memory window becomes apparent once the positive sweep limit exceeds approximately +1.7 V and widens further as the range is increased up to −4.5 → +4.5 V, in agreement with the coercive voltages extracted from the P–V measurement. The ON/OFF current ratio reaches ∼10^5^–10^6^ at a small drain bias (V_sd_ = 50 mV), indicating strong electrostatic control of the monolayer MoS_2_ channel.

Non‐volatile retention of multiple conductance states is essential for inference‐oriented neuromorphic hardware and is demonstrated in Figure 2e. After initializing the device in a low‐conductance state with a −4.0 V gate pulse, different positive programming pulses (2.0, 2.5, 3.0, and 3.5 V) are applied to set distinct channel conductances. The drain current at V_g_ = 0 is then monitored as a function of time. All programmed conductance states remain stable for at least 2 × 10^4^ s without noticeable drift. These results confirm robust non‐volatile memory behavior and provide a solid device foundation for the synaptic operation discussed below.

### 2.3 Near‐Coercive Synaptic Operation and Device‐Informed Crossbar Accuracy

To use the FeFET as a synapse, we operate it in a near‐coercive regime where small programming pulses induce gradual, partial polarization switching [33]. In this mode, trains of potentiation (+3.0 V) or depression (−1.8 V) pulses are applied to the gate, and after each pulse the *I*_d–_ *V*_g_ curve is measured in a narrow gate‐voltage window (−0.6 to 0.5 V) at a small drain bias (Figure 3a). Within this restricted sweep range, additional polarization switching does not occur, so that the pulse‐induced modulation of the channel conductance can be tracked without interference from full ferroelectric reversal. The resulting trajectories show that both P/D produce smooth, nearly uniform conductance changes per pulse.

The microscopic picture underlying this behavior is illustrated schematically in Figure 3b. Successive positive pulses gradually increase the net polarization in the HZO layer and raise the MoS_2_ channel conductance (potentiation), whereas successive negative pulses partially reverse the polarization and lower the conductance (depression). Together, these processes form a reversible P/D cycle in which the device conductance plays the role of the synaptic weight.

The core synaptic behavior is captured by P/D characteristics measured for different pulse counts under identical programming conditions (V_P_/V_D_ = +2.6/−2.08 V, pulse width 500 ms; Figure 3c). For total pulse numbers of 100, 200, 300, and 400 (with equal numbers of P/D pulses in each cycle), the conductance trajectories exhibit a self‐similar shape that scales with pulse count: increasing the number of pulses increases the number of accessible intermediate conductance levels while preserving the overall loop form. Each curve in Figure 3c is an average over many repeated cycles, highlighting that this analog switching behavior is highly reproducible. Cycling measurements for the #200 condition (Figure 3d) further show that the P/D operation is stable over thousands of pulses, with only minor drift in the high and low conductance states, indicating that the device can sustain repeated weight updates during training or calibration.

To quantify the impact of these device characteristics on neural‐network performance, we implemented a device‐informed simulator in which synaptic weights are directly mapped onto the experimentally measured P/D curves (Figure 3e). Using a fully connected 784–300–10 network trained on MNIST for 20 epochs, an ideal digital reference with full‐precision weights achieves an accuracy of approximately 94%. When the same network is constrained to use the FeFET‐derived conductance trajectories, the device‐informed simulator attains an accuracy of about 91% for the #200 pulse condition, with similar values for #300 and #400. Thus, the analog FeFET synapses deliver inference performance that remains close to the ideal baseline, despite finite weight resolution and device non‐idealities.

Write noise plays a key role in this comparison. By contrasting simulations that include the experimentally observed cycle‐to‐cycle variability in conductance with those in which this variability is artificially suppressed, we isolate the effect of write noise (Figure 3e, inset). For the #100 pulse condition, removing cycling‐induced variations significantly improves the simulated accuracy, indicating that write noise is already non‐negligible even in this relatively short P/D sequence (Figure S7). This sensitivity motivates a more detailed, quantitative treatment of write noise and sets the stage for the quantized machine‐learning analysis in Figure 5.

### 2.4 Pulse‐Engineered Modulation Of Weight Dynamic Range And Resolution

Different neuromorphic systems impose different requirements on synaptic weights—for example, in terms of dynamic range, number of effective levels, and available programming time. To map out how the MoS_2–_HZO FeFET can be adapted to these constraints, we systematically modulate the pulse conditions and examine the resulting P/D curves (Figure 4). Conceptually, the design space can be viewed in terms of the conductance dynamic range *G*_max_/*G*_min_ and the Pulse # defining the effective weight levels. By appropriate choice of the amplitudes and widths of P/D pulses, the device can be biased into regimes with large or small dynamic range and with coarse or fine weight resolution, covering the four representative cases sketched in Figure 4a.

Systematic measurements elucidate how pulse parameters control this behavior. When the strengths of P/D are varied together, either by changing the pulse amplitude or the pulse width, both the conductance swing and the granularity of the P/D loop change markedly (Figure 4b‐1,b‐2). Larger or longer pulses switch a greater fraction of ferroelectric domains per event, yielding a wider dynamic range but a coarser conductance granularity at a fixed pulse count, whereas smaller or shorter pulses compress the dynamic range and enable finer granularity.

Varying only the potentiation strength while keeping the depression pulses fixed produces similar trends (Figure 4c‐1,c‐2). Increasing the potentiation amplitude or width steepens the potentiation branch and leads to a faster approach to the high‐conductance state, while reducing these parameters yields a more gradual, nearly linear trajectory. These observations indicate that the potentiation strength largely sets the overall span and level density of the P/D loop, and therefore the achievable weight resolution.

In contrast, varying only the depression strength at fixed potentiation conditions primarily shifts the P/D loop vertically without significantly altering its span (Figure 4d‐1,d‐2). Stronger depression pulses shift the entire loop toward lower conductance, effectively lowering the offset of the accessible window, whereas weaker depression pulses shift it toward higher conductance, raising the offset while leaving the dynamic range nearly unchanged. This behavior is particularly useful when a neuromorphic circuit specifies a limited read‐out range: the same dynamic range can be positioned at different absolute conductance levels by tuning only the depression pulses.

Finally, the results in Figure 4 collectively indicate that the incremental conductance update per pulse (Δ*G*) can be tuned by the effective programming stimulus, set by the pulse amplitude and width. Increasing either parameter strengthens the stimulus and increases Δ*G* per event, whereas reducing them yields smaller, finer updates. Together, Figure 4b–d show that pulse engineering provides a practical set of knobs for tailoring both the dynamic range and the effective weight resolution of the FeFET synapse—without changing the device structure.

### 2.5 Quantized Neural‐Network Modeling: PTQ vs QAT With Device‐Derived Noise

Analog AI hardware inevitably faces physical non‐idealities such as cycle‐to‐cycle write variability, nonlinear update behavior (including the effective step number and nonlinearity of Δ*G*), and array‐level variability (Figure 5a) [34, 35]. While these effects complicate reliable deployment of neuromorphic hardware, QML provides a practical route to make inference robust against such hardware limits. In the following, we compare PTQ and QAT under device‐derived constraints, and show how noise‐aware QAT in particular enables accurate inference on inherently quantized, noisy analog synapses.

The device‐level measurements above naturally lead to a quantized neural‐network description in which each synaptic weight is constrained to a finite set of conductance levels and subject to write noise. To assess how different training strategies cope with these constraints, we performed simulations using a 784–128–64–10 fully connected network on the MNIST task, explicitly incorporating experimentally measured conductance statistics into the model. In all cases, training was limited to 20 epochs to avoid over‐optimizing the algorithm relative to the hardware.

We first compare ideal full‐precision training with quantized models in the absence of noise (noise scaling factor 0×) using 100 conductance levels, corresponding to the typical number of resolvable states in our FeFET (Figure 5b). The ideal digital network with Float32 weights reaches a test accuracy of approximately 97%–98%. A QAT model constrained to 100 levels closely tracks this accuracy, whereas a PTQ model with the same number of levels shows slightly lower and more fluctuating performance. This baseline comparison indicates that QAT can largely compensate for the loss of precision when noise is negligible.

Next, we examine robustness against write noise (Figure 5c). For both PTQ and QAT, write noise is modeled using the experimentally extracted standard deviation of conductance updates, and its magnitude is scaled from 0 × (no noise) to 30 ×; a scaling factor of 1 × corresponds to the measured device noise. At 1× noise, representative of our MoS_2–_HZO FeFET synapses, the QAT model maintains high accuracy close to the noise‐free case, whereas the PTQ model shows noticeable degradation. As noise is further increased to 5 × and 10 ×, the PTQ accuracy drops rapidly, while QAT remains comparatively robust. Only at an extreme 30 × noise level—well beyond the measured device noise—do both methods exhibit severe accuracy loss. These trends indicate that the experimentally observed write noise can be compatible with reliable analog inference, provided it is taken into account during training.

We also vary the number of quantization levels, using values between 100 and 5 to emulate different effective bit depths (Figure 5d). For QAT, the test accuracy remains high and is only weakly dependent on the number of levels and on noise scaling factors up to 5 ×. Even with only 5 conductance levels, the QAT models achieve accuracies close to those of the higher‐precision cases. PTQ, in contrast, shows a systematic downward shift in accuracy as the number of levels is reduced and as noise increases, with the strongest degradation at 5 levels and high noise. This behavior highlights that PTQ is more sensitive to both device noise and reduced precision than QAT. The results in Figure 5c,d show mean accuracies over 35 runs with distinct random seeds, with error bars indicating ±1 standard deviation. The smaller run‐to‐run variability of QAT in Figure 5d further supports its suitability for quantized inference with device‐derived write noise.

Weight histograms provide additional insight into how the models use the available conductance states (Figure 5e–g). For QAT with noise scaling 1 ×, distinct peaks corresponding to individual conductance levels are clearly visible when only 5 or 10 levels are allowed, whereas for 30 levels the peaks broaden and partially overlap (Figure 5e). When the noise scaling is increased at fixed 10‐level quantization, the peaks broaden further and eventually merge into a smoother distribution (Figure 5f), yet the corresponding QAT models still exhibit excellent accuracy, consistent with the robustness in Figure 5d. A direct comparison between PTQ and QAT under the same aggressive conditions (10 levels and 5× noise) shows that their weight histograms are remarkably similar (Figure 5g), even though the QAT model outperforms PTQ by several percentage points in accuracy. This indicates that subtle differences in how weights are assigned to discrete levels during training have a strong impact on performance, and that noise‐aware QAT is essential for fully exploiting quantized, noisy analog hardware based on MoS_2–_HZO FeFET synapses.

## 3 Conclusion

In summary, we realized a monolayer MoS_2–_HZO FeFET as a reconfigurable synaptic transistor that supports gradual, multi‐level potentiation and depression under identical programming pulses [36]. By pairing an ultrathin‐body MoS_2_ channel with a CMOS‐compatible HZO ferroelectric gate stack, the device provides a scaling‐relevant platform for non‐volatile, voltage‐programmable conductance modulation. Through systematic program‐pulse engineering, we showed that the synaptic P/D characteristics can be tuned without changing the device structure: identical voltage pulses produce gradual conductance updates, and adjusting pulse amplitude and width modulates the update step size, dynamic range, and effective linearity of weight trajectories. The resulting large number of discernible conductance states under identical‐pulse operation indicates a simple, array‐compatible route to multi‐level weight storage in crossbar‐scale neuromorphic hardware.

At the system level, we integrated experimentally measured device behavior—including finite conductance levels and cycle‐to‐cycle write variability—into neural‐network inference models to obtain hardware‐consistent performance estimates. This device‐informed approach shows that write noise is a critical constraint: if it is averaged out, accuracy can be overestimated, whereas explicitly retaining it enables realistic evaluation of inference robustness. Within a quantized machine‐learning framework, we further compared post‐training quantization (PTQ) with noise‐aware quantization‐aware training (QAT) under device‐derived quantization and write‐noise conditions. QAT consistently preserved accuracy more effectively than PTQ in low‐precision and noisy regimes, establishing a practical co‐design principle: robust inference on inherently quantized, noisy analog synapses requires hardware‐consistent, noise‐aware training [37]. Together, these results bridge pulse‐level ferroelectric programming physics and noise‐robust quantized inference, highlighting monolayer MoS_2–_HZO FeFET synapses as a promising path toward energy‐efficient, scalable edge‐AI and neuromorphic computing systems [38].

## 4 Experimental Section/Methods

### 4.1 Device Fabrication

The monolayer MoS_2–_HZO FeFET devices were fabricated on a silicon substrate with a 300 nm SiO_2_ insulating layer. A bottom gate electrode of titanium nitride (TiN, ∼200 nm) was first deposited on the substrate, serving both as the bottom gate electrode and as the growth substrate for HZO [39]. Next, a ∼10 nm HZO thin film was deposited on the TiN by thermal atomic layer deposition (ALD) using cyclopentadienyl‐tris(dimethylamino)‐hafnium (Hf[Cp(NMe_2_)_3_]) and cyclopentadienyl‐tris(dimethylamino)‐zirconium (Zr[Cp(NMe_2_)_3_]) precursors with ozone at a deposition temperature of 320°C [40].

Subsequently, a CVD‐grown monolayer MoS_2_ was transferred onto the HZO/TiN/SiO_2_ stack via a wet transfer process using a KOH solution [41]. After transfer, source and drain (S/D) contacts were patterned on the MoS_2_ by electron‐beam lithography, followed by the deposition of In/Au (10/40 nm) contacts by e‐beam evaporation. The channel length was defined to be ∼1 µm, while the channel width was determined by the lateral size of the MoS_2_ monolayer. The resulting device is a bottom‐gated FeFET, in which the TiN layer serves as the bottom gate electrode, HZO acts as the ferroelectric gate dielectric, and the monolayer MoS_2_ functions as the channel layer.

### 4.2 Material and Device Characterization

We performed Raman spectroscopy on the MoS_2_ to verify its layer number and quality. The Raman spectrum (excitation 532 nm) showed the characteristic E^1^_2g_ and A_1g_ peaks at ∼384 and ∼403 cm^−1^, respectively, with a separation of ∼19 cm^−1^, confirming that the MoS_2_ is monolayer (in few‐layer MoS_2_ this peak separation would be larger) [42]. For the HZO ferroelectric layer, GIXRD was used. The crystallinity of the HZO film was examined using a Cu‐Kα radiation source (λ = 1.5405 Å) at an incidence angle of 3°, with 2θ scans collected from 20° to 70°. A prominent diffraction peak is observed around 2θ ≈ 30°–31°, which is commonly consistent with orthorhombic‐related polar phases of HZO [43]. These results indicate the formation of a crystalline HZO phase consistent with ferroelectric HZO films used in device structures.

To verify the ferroelectric behavior of HZO, polarization–voltage (P–V) measurements were performed on a separate metal–ferroelectric–metal capacitor with the same 10 nm HZO layer. Clear polarization hysteresis loops were observed as the voltage sweep range increased, confirming the ferroelectric nature of HZO. The coercive voltages were approximately +2.5 V and –1.7 V, with a slight shift toward positive bias, indicative of built‐in asymmetry in the ferroelectric stack [44]. The voltage range over which polarization switching occurs in this capacitor closely matches the gate‐voltage range where hysteresis appears in the FeFET transfer characteristics, directly linking the ferroelectric capacitor behavior to the device operation.

### 4.3 Basic Electrical Characterization

Basic electrical characterization of the FeFET was carried out using a probe station at room temperature under vacuum conditions (<10^−5^ Torr). The transfer characteristics (*I*_d–_ *V*_g_) were measured by sweeping the bottom‐gate voltage (*V*_g_) applied to the TiN gate electrode while applying a constant drain bias (V_sd_ = 50 mV) and monitoring the drain current (*I*_d_). A counterclockwise hysteresis was observed in the *I*_d–_ *V*_g_ sweep, which is characteristic of general FeFET operation. The hysteresis behavior indicates a gate‐voltage‐dependent modulation of the channel conductance associated with polarization switching in the HZO layer. A discernible memory window appears for gate‐voltage sweeps exceeding ±1.7 V and becomes more pronounced for sweeps up to ±4.5 V. The ON/OFF current ratio of the transistor reaches approximately 10^6^, indicating effective gate control of the MoS_2_ channel.

Additionally, partial polarization switching was induced by gate voltage pulses applied near the coercive voltage of the FeFET. The resulting conductance modulation was examined by performing *I*_d–_ *V*_g_ sweeps within a narrow gate‐voltage range that avoids any further polarization change, enabling observation of pulse‐induced channel conductance changes. This near‐coercive operation forms the basis of the analog weight modulation employed in this work.

### 4.4 Synaptic Programming Protocol

To emulate synaptic weight updates, we applied trains of voltage pulses to the gate and monitored the channel conductance after each pulse at V_G_ = 0. For all synaptic measurement sequences, the device was preset to a low‐conductance state by applying a negative gate‐pulse to initialize the ferroelectric polarization, and subsequently a sequence of identical pulses was applied. The channel conductance *G* thus serves as the synaptic weight, and its incremental changes correspond to potentiation (increase in weight) or depression (decrease in weight).

One P/D cycle is defined as a sequence of potentiation pulses followed by depression pulses, intended to restore the channel conductance close to its initial value. Potentiation pulses were applied with positive gate voltages to increase channel conductance, whereas depression pulses were applied with negative gate voltages to reduce conductance. Various pulse‐number combinations were employed, including 50/50, 100/100, 150/150, and 200/200 pulses, to systematically examine how the shape and resolution of the P/D curves evolve with pulse count.

In addition to pulse number, pulse amplitude and pulse width were also important control parameters. For pulse amplitude, measurements were performed by either fixing the potentiation‐to‐depression voltage ratio or by fixing one polarity (potentiation or depression) while varying the opposite polarity, allowing us to assess how P/D asymmetry influences device behavior. Pulse widths were varied from 200 to 1000 ms, using both symmetric conditions (identical widths for P/D) and asymmetric conditions in which only one pulse type was modified. These measurements were designed to clarify the respective roles of pulse number, amplitude, and width in synaptic weight modulation.

### 4.5 Write Noise Characterization

Write noise was characterized by repeatedly applying identical programming pulses under the same conditions and analyzing the statistical variation in conductance updates. The distribution of pulse‐induced conductance changes was examined using cumulative distribution functions (CDFs) constructed as a function of the conductance state. Write noise was quantified using a normalized programming signal‐to‐noise metric defined as *Δ*G^2^/σ^2^, where *Δ*G and σ are the mean and standard deviation of the per‐pulse conductance increment evaluated in a state‐dependent manner. This analysis provides direct insight into how write noise translates into weight uncertainty and affects inference accuracy in analog neural‐network operation. The observed behavior was consistent across various operating conditions.

### 4.6 Neural Network and Crossbar Simulation

To evaluate the impact of experimentally measured device characteristics on neural network performance, we performed simulations using the MNIST dataset, which consists of 28 × 28 grayscale images of handwritten digits (0–9). A fully connected neural network with a 784–300–10 architecture was employed for crossbar‐level simulations, and training was limited to 20 epochs to avoid excessive algorithmic optimization [45]. As an ideal reference, a purely digital software model with full‐precision weights achieved an inference accuracy of approximately 94%.

We then implemented a crossbar‐level simulator in which synaptic weights were mapped directly onto the experimentally measured P/D conductance characteristics of the FeFET devices [46]. In this framework, synaptic updates follow the measured device behavior, with write noise implicitly inherited from pulse‐to‐pulse conductance variations. In the synaptic‐array model, input activations are applied as gate‐line voltages and the resulting drain currents are summed along bit lines to implement VMM in a crossbar‐like 1T array abstraction. Using this device‐informed crossbar simulator, an inference accuracy of approximately 91% was achieved, which remains close to the ideal software baseline.

To assess how quantization strategy and write noise influence device‐based inference more generally, we conducted additional simulations using a 784–128–64–10 network architecture. In this framework, two weight‐mapping strategies were considered: PTQ and QAT. In PTQ, the model was trained in software and then directly mapped onto discrete conductance levels. In QAT, quantization and write noise were incorporated during training using a straight‐through estimator, enabling the model to adapt to hardware‐induced uncertainty; under these conditions, the QAT‐based simulator achieved an inference accuracy of approximately 97%, essentially matching the ideal full‐precision baseline [47]. We use the 784–300–10 network for direct device‐trajectory mapping and the 784–128–64–10 network for systematic PTQ/QAT sweeps; both are trained for 20 epochs for consistent comparison. For the QAT/PTQ simulations, training used the Adam optimizer with a learning rate of 0.001, a batch size of 64, and the sparse categorical cross‐entropy loss. The comparisons in Figure 5c,d were repeated using 35 distinct random seeds with results reported as mean test accuracy ±1 standard deviation.

For both PTQ and QAT, write noise was modeled using the experimentally extracted conductance standard deviation, and its magnitude was systematically scaled from 0 × (noise‐free), to 1 × (corresponding to the measured device noise), and further increased up to 30 × to emulate increasingly non‐ideal programming conditions. Training and inference accuracy were evaluated across this noise range to quantify the robustness of each approach.

## Author Contributions

**Jeongmin Park**: conceptualization, methodology, investigation, formal analysis, data curation, visualization, writing – original draft. **Moonyoung Jung**: conceptualization, methodology, investigation, resources, writing – original draft. **Hyo‐Bae Kim**: methodology, resources. **Chanhyuk Park**: software, validation, formal analysis. **Seohyun Lee**: investigation, data curation. **Ki Kang Kim**: resources, validation. **Hongkee Yoon**: conceptualization, software, validation, supervision. **Ji‐Hoon Ahn**: methodology, resources, supervision. **Dongseok Suh**: conceptualization, methodology, supervision, project administration, writing – review and editing.

## Conflicts of Interest

The authors declare no conflicts of interest.

## Supporting information




**Supporting File**: advs78366‐sup‐0001‐SuppMat.docx.

## Data Availability

The data that support the findings of this study are available in the supplementary material of this article.
