# Supplementary material for: Quantized Analog Inference Hardware Enabled by MoS2–Hf1‐xZrxO2 FeFET Synapses
Source: Adv Sci (Weinh). 2026 Oct 22:e78366. Online ahead of print. doi: 10.1002/advs.78366 (PMC13690976; doi:10.1002/advs.78366)
Supplement: Supplementary file 1 [file ADVS-9999-e78366-s001.docx]

Supporting Information

Quantized analog inference hardware enabled by MoS_2_–Hf_1-x_Zr_x_O_2_ FeFET synapses

\

*Jeongmin Park†, Moonyoung Jung†, Hyo-Bae Kim, Chanhyuk Park, Seohyun Lee, Ki Kang Kim, Hongkee Yoon*, Ji-Hoon Ahn* & Dongseok Suh**

**Figure S1** | Optical microscope images of fabricated MoS_2_–HZO FeFET devices. Representative optical micrographs of MoS_2_–HZO FeFET fabricated for reproducibility and device-to-device variation studies. More than 10 devices with the same structure were additionally fabricated and electrically tested, and the device-to-device characteristics (for S1~10) are summarized in Figure S4. The device stack is identical to that used in the main text, consisting of a ~200 nm TiN bottom gate, a ~10 nm HZO ferroelectric gate dielectric, and a CVD-grown monolayer MoS_2_ channel.

**Figure S2** | Gate-current characteristics during the asymmetric $V_{g}$sweep used in Figure 2d. $I_{g}$–$V_{g}$ curves measured from the same MoS_2_–HZO FeFET as in Figure 2d ($V_{d}$ = 50 mV). For each trace, the sweep starts at $V_{g}=-4.5$ V, is swept to a turning voltage $V_{g,\mathrm{turn}}$(legend), and then returns to $-4.5$ V (arrows indicate the sweep sequence). The evolution of $I_{g}$with $V_{g,\mathrm{turn}}$, including pronounced features near the switching voltages, reflects ferroelectric polarization switching in the HZO gate stack.

**Figure S3-1** | $I_{d}$–$V_{g}$ transfer characteristics of MoS_2_–HZO FeFET measured with asymmetric sweeps starting from opposite preset biases ($V_{d}=50$ mV). Blue: the device was first preset at $V_{g}=-4.5$ V, then $V_{g}$was swept toward positive bias to a series of progressively larger positive turning voltages (top arrow sequence) and swept back to $-4.5$ V, producing the overlaid hysteresis loops. Red: the device was preset at $V_{g}=+4.5$ V, then $V_{g}$was swept toward negative bias to progressively larger-magnitude negative turning voltages (bottom arrow sequence) and returned to $+4.5$ V. Notably, little to no hysteresis is observed when the turning voltage remains within the sub-coercive regime; a pronounced memory window emerges once the sweep exceeds the coercive voltage, consistent with polarization switching–driven modulation of channel conductance in a ferroelectric field-effect transistor.

**Figure S3-2** | $I_{d}$–$V_{g}$ transfer characteristics measured with symmetric gate-voltage sweeps ($V_{d}=50$ mV). The gate voltage was swept between $-V_{g,\max}$and $+V_{g,\max}$and back (arrows), while the sweep range was progressively expanded (different colors). The hysteresis (memory window) increases as the sweep exceeds the coercive-voltage regime, consistent with polarization switching in the HZO gate stack governing the FeFET conductance.

**Figure S4 | Device-to-device variation of MoS_2_–HZO FeFET synaptic devices.** Overlaid $I_{g}$–$V_{g}$ (top) and geometry-normalized $I_{d}$–$V_{g}$ transfer characteristics (bottom, log scale) measured from 10 devices fabricated with the same structure ($V_{d}=50$ mV). For the bottom panel, the drain current was normalized by the channel geometry as $I_{d}L/W$, where $W$and $L$are the channel width and length, respectively. The devices shown in Figure S1 exhibit similar ferroelectric hysteresis (memory-window) characteristics, with modest variations in the normalized ON current and subthreshold behavior; small $I_{g}$spikes appear near the highest positive $V_{g}$in some devices.

**Figure S5** | Transistor and memory characteristics of a MoS_2_–HZO FeFET. (a) $I_{d}$–$V_{g}$ transfer curve measured at $V_{d}=50$ mV during a full sweep from $-5$ to $+5$ V (and back), showing a clear ferroelectric hysteresis window. (b) Corresponding gate current ($I_{g}$–$V_{g}$) recorded simultaneously, plotted in the pA range; distinct $I_{g}$ features become more pronounced at high positive $V_{g}$, consistent with polarization-switching–related processes in the HZO gate stack. (c) Subthreshold swing (SS) extracted as a function of drain current $I_{d}$ for the increasing-$V_{g}$ and decreasing-$V_{g}$ sweep directions, reaching $\lesssim100$ mV/dec in the subthreshold regime. (d) Cycling endurance under alternating program/erase gate pulses ($V_{g}=+4$ V and $-4$ V), demonstrating reproducible switching of $I_{d}$ between high- and low-conductance states over $>700$ cycles.

**Figure S6** | Raw potentiation/depression (P/D) characteristics under pulse-engineering conditions (source data for Figure 3c–e and Figure 4b–d). All data are raw measurement results acquired by repeatedly applying an identical P/D pulse sequence for each condition, accumulating >5000 total pulses, thereby capturing cycle-to-cycle variation and write noise. In each subpanel, the left plot shows the conductance evolution $G$ versus cumulative pulse number over repeated P/D cycles, while the right plot shows a representative single-cycle P/D curve from the same dataset.

(a) P/D characteristics measured while varying the voltage amplitude with a fixed ratio ($\mid V_{P}\mid:\mid V_{D}\mid=1:0.8$).
(b) P/D characteristics measured while varying the pulse width symmetrically ($W_{P}=W_{D}=250,$ $500,$ and $750$ ms) at fixed pulse amplitudes.
(c) Potentiation-voltage dependence: $V_{P}$ varied from 2.5 to 3.0 V with fixed $V_{D}=-2.08$ V and fixed pulse widths $W_{P}=W_{D}=500$ ms.
(d) Potentiation pulse-width dependence: $W_{P}$ varied from 250 to 1000 ms with fixed $V_{P}=2.6$ V, $V_{D}=-2.08$ V, and fixed $W_{D}=500$ ms.
(e) Depression-voltage dependence: $V_{D}$ varied from $-1.6$ to $-2.08$ V with fixed $V_{P}=2.6$ V and fixed pulse widths $W_{P}=W_{D}=500$ ms.
(f) Depression pulse-width dependence: $W_{D}$ varied from 250 to 1000 ms with fixed $V_{P}=2.6$ V, $V_{D}=-2.08$ V, and fixed $W_{P}=500$ ms.
(g) P/D characteristics measured with different pulse numbers per P/D cycle (pulse number = 100–500) at fixed pulse amplitudes.
(h) Complete raw P/D dataset obtained from a single MoS_2_–HZO FeFET during a continuous measurement, showing the conductance evolution as a function of cumulative pulse number over the entire pulse-engineering sequence.

**Figure S7** | Long-run conductance evolution under repeated P/D cycling with different pulse numbers per cycle. Conductance was recorded as a function of cumulative pulse number while repeatedly applying identical P/D pulse trains at $V_{P}=+2.6$V and $V_{D}=-2.08$V with a pulse width of 500 ms. Two protocols are compared: #100 (50 potentiation + 50 depression pulses per cycle, blue) and #200 (100 potentiation + 100 depression pulses per cycle, red). The traces show the accumulated P/D cycling response over thousands of pulses, illustrating how increasing the number of pulses per cycle enlarges the conductance excursion during each P/D update.

**Figure S8** | Pulse-engineering dependence of synaptic metrics and inference accuracy

Figure S8-1–S8-7 summarize how programming-pulse conditions shape the potentiation/depression (P/D) behavior of the MoS₂–HZO FeFET synapse and how the extracted device metrics translate into inference accuracy in device-informed MNIST simulations (corresponding to the parameter sweeps used for Figure 3c–e and Figure 4b–d in the main text). Unless noted otherwise, conductance trajectories include cycle-to-cycle variation (write noise) without post-averaging; thus, the plots represent measured variability rather than idealized updates. The absolute MNIST accuracy depends on the simulator settings (network/training recipe), so these results are primarily intended for relative comparison across pulse conditions.

Panel definitions (common to S8-1–S8-7):
(a) Update nonlinearity for potentiation and depression extracted from the measured P/D trajectories.
(b) MNIST inference accuracy obtained using the measured update rules.
(c) Conductance window (G_max_ and G_min_) as a function of the swept parameter.
(d) Dynamic range (G_max_/G_min_) as a function of the swept parameter.
(e) Representative single-cycle P/D curve (conductance vs pulse index) for selected conditions.
(f,g) ΔG–G CDF (cumulative distribution function) maps (i.e., conductance-update statistics; write-noise characteristics) for the lowest-accuracy and highest-accuracy conditions in each sweep, respectively (conditions explicitly stated below).

**Figure S8-1** | Voltage-range dependence at a fixed P/D amplitude ratio

P/D amplitudes were swept while keeping the magnitude ratio (|V_P_|:|V_D_| = 1:0.8) with fixed pulse widths W_P_ = W_D_ = 500 ms. Panels (a–e) follow the common definitions above. Panels (f,g) show ΔG–G CDF maps for Voltage range = 4.68 V (Accuracy = 87%; lowest) and Voltage range = 5.22 V (Accuracy = 89%; highest), respectively.

**Figure S8-2** | Pulse-width (W_P_ = W_D_) dependence

Pulse width was varied (W_P_ = W_D_ = 250–750 ms) at fixed pulse amplitudes V_P_ = 2.60 V and V_D_ = -2.08 V. Panels (a–e) follow the common definitions above. Panels (f, g) show ΔG–G CDF maps for the lowest-accuracy case (Accuracy = 88.5%) and the highest-accuracy case (Accuracy = 89.2%), as indicated in the figure annotations.

**Figure S8-3** | Potentiation-voltage (V_P_) dependence

Potentiation amplitude was swept (V_P_ = 2.5–3.0 V) at fixed V_D_ = -2.08 V and fixed pulse widths W_P_ = W_D_ = 500 ms. Panels (a–e) follow the common definitions above. Panels (f, g) show ΔG–G CDF maps for V_P_ = 2.5 V (Accuracy = 84%; lowest) and V_P_ = 3.0 V (Accuracy = 89%; highest), respectively.

**Figure S8-4** | Potentiation pulse-width (W_P_) dependence

Potentiation pulse width was swept (W_P_ = 200–1000 ms) at fixed V_P_ = 2.60 V and V_D_ = -2.08 V, with depression width fixed at W_D_ = 500 ms. Panels (a–e) follow the common definitions above. Panels (f, g) show ΔG–G CDF maps for W_P_ = 500 ms (Accuracy = 88.5%; lowest) and W_P_ = 1000 ms (Accuracy = 89.5%; highest), respectively.

**Figure S8-5** | Depression-voltage (V_D_) dependence

Depression amplitude was swept (V_D_ = -1.60 to -2.08 V) at fixed V_P_ = 2.60 V and fixed pulse widths W_P_ = W_D_ = 500 ms. Panels (a–e) follow the common definitions above. Panels (f, g) show ΔG–G CDF maps for V_D_ = -1.90 V (Accuracy = 87.5%; lowest) and V_D_ = -1.60 V (Accuracy = 89.5%; highest), respectively.

**Figure S8-6** | Depression pulse-width (W_D_) dependence

Depression pulse width was swept (W_D_ = 200–1000 ms) at fixed V_P_ = 2.60 V and V_D_ = -2.08 V, with potentiation width fixed at W_P_ = 500 ms. Panels (a–e) follow the common definitions above. Panels (f, g) show ΔG–G CDF maps for W_D_ = 200 ms (Accuracy = 87%; lowest) and W_D_ = 1000 ms (Accuracy = 89%; highest), respectively.

**Figure S8-7** | Pulse-number dependence

The number of pulses per potentiation/depression segment was varied (pulse # = 100–400) at fixed V_P_ = 2.60 V, V_D_ = -2.08 V, and W_P_ = W_D_ = 500 ms. Panels (a–e) follow the common definitions above. Panels (f, g) show ΔG–G CDF maps for pulse # = 100 (Accuracy = 85%; lowest) and pulse # = 200 (Accuracy = 91%; highest), respectively.

**Figure S9 | Potentiation/depression characteristics of additional MoS_2_–HZO FeFET devices.** (a,b) Pulse-number dependence measured from an additional device. (a) Conductance evolution over repeated P/D cycles under different pulse-number conditions. (b) P/D curves averaged over the repeated cycles shown in (a). (c,d) Pulse-width dependence measured from a second additional device. (c) Conductance evolution over repeated P/D cycles for different programming-pulse widths. (d) P/D curves averaged over the repeated cycles shown in (c). The additional devices exhibit gradual and systematic P/D modulation and retain the expected dependence on pulse number and pulse width, providing qualitative support for the reproducibility of pulse-engineered synaptic operation across devices.

**Table S1 |** Quantitative comparison of representative HZO-based ferroelectric synaptic transistors. The device and channel materials, ferroelectric layer, potentiation/depression (P/D) programming scheme, representative pulse conditions, number of accessible conductance states, conductance dynamic range, and neural-network performance are summarized. Values are taken from the corresponding reports. Because the network architectures, training protocols, and device-to-algorithm mapping methods differ among the studies, the reported neural-network accuracies are provided for contextual comparison rather than as a direct head-to-head benchmark.

**References**

1.	Radisavljevic, B., Radenovic, A., Brivio, J., Giacometti, V. & Kis, A. “Single-layer MoS_2_ transistors”. *Nature Nanotechnology* (2011) **6**, 147–150.

2.	Lee, C., Yan, H., Brus, L. E. *et al.* “Anomalous lattice vibrations of single- and few-layer MoS_2_”. *ACS Nano* (2010) **4**, 2695–2700.

3.	Cha, M.-Y., Liu, H., Wang, T. *et al.* “MoS_2_-based ferroelectric field-effect transistor with atomic layer deposited Hf_0.5_Zr_0.5_O_2_ films toward memory applications”. *AIP Advances* (2020) **10**, 065107.

4.	Jiao, H., Wang, X., Wu, S., Chen, Y. & Wang, J. “Ferroelectric field effect transistors for electronics and optoelectronics”. *Applied Physics Reviews* (2023) **10**, 011310.

5.	Mulaosmanovic, H., Breyer, E. T., Dünkel, S. *et al.* “Ferroelectric field-effect transistors based on HfO_2_: a review”. *Nanotechnology* (2021) **32**, 502002.

6.	Böscke, T. S., Müller, J., Bräuhaus, D., Schröder, U. & Böttger, U. “Ferroelectricity in hafnium oxide thin films”. *Applied Physics Letters* (2011) **99**, 102903.

7.	Müller, J., Böscke, T., Braeuhaus, D. *et al.* “Ferroelectric Zr_0.5_Hf_0.5_O_2_ thin films for nonvolatile memory applications”. *Applied Physics Letters* (2011) **99**, 112901.

8.	Sebastian, A., Le Gallo, M., Khaddam-Aljameh, R. & Eleftheriou, E. “Memory devices and applications for in-memory computing”. *Nature Nanotechnology* (2020) **15**, 529–544.

9.	Xiang, H., Chien, Y. C., Li, L. *et al.* “Enhancing memory window efficiency of ferroelectric transistor for neuromorphic computing via two‐dimensional materials integration”. *Advanced Functional Materials* (2023) **33**, 2304657.

10.	Lee, Y., Goh, Y., Hwang, J., Das, D. & Jeon, S. “The influence of top and bottom metal electrodes on ferroelectricity of hafnia”. *IEEE Transactions on Electron Devices* (2021) **68**, 523–528.

11.	Kim, H.-B., Jung, M., Oh, Y. *et al.* “Superior and stable ferroelectric properties of hafnium-zirconium-oxide thin films deposited via atomic layer deposition using cyclopentadienyl-based precursors without annealing”. *Nanoscale* (2021) **13**, 8524–8530.

12.	Tsai, S.-H., Fang, Z., Wang, X. *et al.* “Stress-memorized HZO for high-performance ferroelectric field-effect memtransistor”. *ACS Applied Electronic Materials* (2022) **4**, 1642–1650.

13.	Kim, M. K. & Lee, J. S. “Ferroelectric analog synaptic transistors”. *Nano Letters* (2019) **19**, 2044–2050.

14.	Halter, M., Bégon-Lours, L., Sousa, M. *et al.* “A multi-timescale synaptic weight based on ferroelectric hafnium zirconium oxide”. *Communications Materials* (2023) **4**, 14.

15.	Byun, K., Choi, I., Kwon, S. *et al.* “Recent advances in synaptic nonvolatile memory devices and compensating architectural and algorithmic methods toward fully integrated neuromorphic chips”. *Advanced Materials Technologies* (2023) **8**, 2200884.

16.	Xiao, T. P., Feinberg, B., Bennett, C. H. *et al.* “On the accuracy of analog neural network inference accelerators”. *IEEE Circuits and Systems Magazine* (2023) **22**, 26–48.

17.	Jacobs-Gedrim, R. B., Agarwal, S., Knisely, K. E. *et al.* “Impact of linearity and write noise of analog resistive memory devices in a neural algorithm accelerator”. in Proceedings of the *2017 IEEE International Conference on Rebooting Computing (ICRC)* (2017) 1–10.

18.	Sun, X. & Yu, S. “Impact of non-ideal characteristics of resistive synaptic devices on implementing convolutional neural networks”. *IEEE Journal on Emerging and Selected Topics in Circuits and Systems* (2019) **9**, 570–579.

19.	Kim, M.-K., Kim, I.-J. & Lee, J.-S. “Oxide semiconductor-based ferroelectric thin-film transistors for advanced neuromorphic computing”. *Applied Physics Letters* (2021) **118**, 032902.

20.	Xi, F., Han, Y., Grenmyr, A., Grützmacher, D. & Zhao, Q.-T. “Four-Terminal Ferroelectric Schottky Barrier Field Effect Transistors as Artificial Synapses for Neuromorphic Applications”. *IEEE Journal of the Electron Devices Society* (2022) **10**, 569–574.
